# The implications of alcohol mixed with energy drinks from medical and socio-legal standpoints

**DOI:** 10.3389/fnbeh.2022.968889

**Published:** 2022-11-09

**Authors:** Jessica Atef Nassef Sefen, Jayaditya Devpal Patil, Helen Cooper

**Affiliations:** Royal College of Surgeons in Ireland – Bahrain,, Muharraq, Bahrain

**Keywords:** alcohol, energy drinks, caffeine, taurine, alcohol mixed with energy drink (AMED), adenosine, dopamine, regulations

## Abstract

Co-ingestion of energy drinks and alcohol has long been in practice and has been poorly regulated despite a growing body of literature of their potential negative health impacts. Co-ingestion of energy drinks with alcohol has multiple counter-active effects such as reduction of body sway, fatigue and sedative effects induced by alcohol, along with increased subjective feeling of alertness, which may lead to increased binge-drinking, intoxication, decreased perception of intoxication, dehydration, and alcohol poisoning. Adding energy drinks to alcohol may also have synergistic effects in causing alcohol dependency and addiction. The association between caffeine, a common active ingredient in energy drinks, and alcohol is relatively well defined, however association with other active ingredients such as taurine, niacin, and pyridoxine, is less understood, pointing to a gap in our knowledge regarding this practice. Nonetheless, the current associations between AMED (Alcohol Mixed with Energy Drinks) and risky behavior secondary to intoxication and cases of alcohol poisoning have led various national governing bodies to regulate this practice. This review highlights the potential effects of AMED on human physiology based on what is known from human and animal models, and sheds light on specific biochemical interactions between alcohol and active ingredients found in energy drinks; Caffeine, Taurine, and Glucuronolactone. The review also touches on the regulation of this practice around the world, and the impact it has on its users, and points researchers to gaps in our knowledge on the interactions between alcohol and EDs and the full extent of their effects.

## Introduction

### Alcohol mixed with energy drinks; epidemiology

The co-ingestion of alcohol mixed with energy drinks (AMED) is a very common practice worldwide, particularly prominent in the younger population where studies from the United States (US) revealed 10.6% of students in grades 8, 10, and 12, and 31.8% of young adults aged 19–28 had consumed AMED at least once in the past year (Kponee et al., 2014; Johnson et al., 2018; Schulenberg et al., 2018). A study surveying college students in the US regarding patterns of energy drink (ED) use found the consumption of AMED was reported by 57% of women and 50% of men (Malinauskas et al., 2007). Another survey of 450 medical students in Italy, 56.9% reported ED consumption of which 48.4% mixed EDs with alcohol; 36% of those who reported ever combining ED and alcohol had done so on more than three occasions in the previous month (Oteri et al., 2007). Similar figures have been found in research investigating the frequency of ED usage among college students in Turkey, where 37.2% reported mixing EDs with alcohol (Attila and Çakir, 2011). Although unclear why young drinkers are driven to consume AMEDs, some literature suggests consumption during parties, taste enhancement of alcohol, and energy and pleasure seeking behaviors are some motivators (Ballistreri and Corradi-Webster, 2008; O’Brien et al., 2008). Regardless of the motives, this phenomenon of mixing energy drinks with alcohol is quite prevalent in today’s society, although unclear if the consumption patterns have increased over time. It is thus important to establish their combinatorial effects and potential implications–which are vital to understand from both medical and sociolegal perspectives–such as drinking habits among users, addiction, binge-drinking, alcohol poisoning, and general wellbeing. This review aims to highlight the aforementioned literature on AMED, while also shedding light on specific interactions between alcohol and active ingredients found in EDs, the impact it has on human physiology and safety, and existing regulations on EDs in different parts of the world.

### Biochemical interactions between alcohol and active ingredients in energy drinks

Alcohol is known to potentiate GABAergic neurotransmission by increasing GABA release (Kumar et al., 2009; Woodward, 2009). In addition, alcohol inhibits glutamatergic neurotransmission by acting on ionotropic glutamate receptors (Siggins et al., 2003; Woodward, 2009). Alcohol also modulates dopamine neurotransmission by directly altering the activity of dopamine neurons in the ventral tegmental area (VTA) (Morikawa and Morrisett, 2010). It is widely believed that alcohol-induced increase in VTA neuronal activity leads to increased dopamine release which mediates the reinforcing euphoric effects of alcohol (Gonzales et al., 2004; Tupala and Tiihonen, 2004). This mechanism may be affected by active ingredients found in EDs.

#### Caffeine-alcohol interactions

The most common ingredient in EDs is caffeine, which is often combined with taurine, glucuronolactone, and B vitamins such as niacin, and pyridoxine (Reissig et al., 2009). Caffeine is a non-selective competitive antagonist at the adenosine receptor and produces its effects by counteracting the effects of endogenous adenosine (O’Brien, 2009). Adenosine is an inhibitory neurotransmitter that increases sedation and contributes to sleep under normal conditions, hence these inhibitory effects of caffeine induces wakefulness. This interaction relies on the ability of adenosine to modulate the function of central ascending neurotransmitter systems, which are involved in motor activation and reward dopaminergic systems and arousal effects through cholinergic, noradrenergic, histaminergic, and orexinergic systems. Among the four A_1_, A_2A_, A_2B_, and A_3_ adenosine receptors, A_1_ and A_2A_ receptors are the ones predominantly expressed in the brain and are the primary target receptors for caffeine (Fredholm et al., 1999; Ferré, 2010; Juliano et al., 2014). The arousing effects of caffeine depend on the inhibition of multiple inhibitory mechanisms that adenosine exerts on (Ferré, 2010). As dopamine activity is involved in the reward system, elevated dopamine levels play a key role in the abuse potential of most drugs of abuse, including alcohol. The activation of adenosine receptors inhibits dopamine activity through proposed mechanisms including A_2A_/D2 and A_1_/D1 receptor-receptor interaction (Ferré et al., 2008; Ferré, 2010) and modulation of dopamine binding affinity (Franco et al., 2000), therefore caffeine indirectly increases dopamine activity. Because caffeine acts as an adenosine receptor antagonist, it blocks adenosine activity, resulting in increased dopamine activity (Nagy et al., 1990; Garrett and Griffiths, 1997; Yao et al., 2002). These biochemical interactions create the basis of the claim that AMED may be dangerous because the stimulant effects of caffeine counteract the sedative effects of alcohol, giving users a false feeling of sobriety thus increasing alcohol consumption and encouraging risk-taking behavior (such as driving under the influence of alcohol). The increased dopamine also stimulates the brain reward system when alcohol is mixed with it, thus the striatal A_2A_–D_2_ receptor interactions provide an important pathway by which caffeine can potentiate the reinforcing euphoric effects of alcohol.

The role of adenosine antagonism on voluntary ethanol intake was explored in literature examining the effect of different caffeine doses and selective adenosine A_1_ and A_2A_ receptor antagonists in mice. This study found caffeine between doses of 2.5–20.0 mg/kg to significantly increase ethanol consumption in moderate ethanol consumers, however no effects were observed in low or high ethanol consumers (SanMiguel et al., 2019).

A preclinical study that assessed the effect of pre-treatment with caffeine on voluntary ethanol consumption in rodents found low and high caffeine doses to have no effect on ethanol consumption but moderate doses of 5 mg/kg to increase ethanol intake (Kunin et al., 2000). A second animal study assessing if caffeine would enhance ethanol-conditioned place preference and enhance ethanol-stimulated locomotor activity found higher doses of caffeine to result in statistically significant increase in locomotion, however, at higher doses of alcohol this is decreased. No significant findings were found in terms of place preference (Hilbert et al., 2013). One trial assessing the interaction between caffeine and ethanol to assess if caffeine can affect the ability of ethanol to elicit conditioned place preference and conditioned place aversion, found caffeine to not have an effect on its own, while ethanol elicited significant conditioned-place preference and aversion. Caffeine significantly prevented ethanol-elicited conditioned-place preference and, also prevented the acquisition of ethanol-elicited conditioned-place aversion (Porru et al., 2020).

Increased drinking with EDs may be explained through the A_2A_ receptor mechanism wherein activation of the A_2A_ receptor decreases alcohol consumption (Houchi et al., 2008) and caffeine is known to inhibit this receptor, therefore potentially attributing to increased alcohol intake (Ferré, 2010).

Alcohol mixed with energy drinks has been shown to increase binge-drinking in some animal studies due to interactions between shared receptors and proposed that such interaction may have a dose dependent relationship (SanMiguel et al., 2019). However this does not mean the same is also applicable to humans.

In another instance of a study looking at the interaction of caffeine on the effects of alcohol on conditioned taste aversion, ataxia, and locomotor activity in mice found that the combination of caffeine and alcohol produced robust locomotor sensitization. After repeated exposure, the effect of the drug combination on activity was approximately 2.5 times greater than either that produced by alcohol or caffeine alone. The authors conclude that co-intoxication with caffeine and alcohol has a possible synergistic effect on locomotor sensitization rather than an additive one. The study revealed that previous caffeine exposure increased the ataxic response to the caffeine and alcohol combination, however, seemed to reduce the ataxic response to high doses of alcohol. The artical failed to show any effect on conditioned taste aversion (Christina et al., 2015). An additional study looking at how ED affects the expression of ethanol sensitization in mice indicated that an alcohol sensitization effect could be enhanced in mice when acutely challenged with a mixture of an ED and alcohol (Ferreira et al., 2013). In summary, these animal model studies indicate that a history of exposure to the combination of alcohol and energy drinks may influence the locomotor response when subjects are acutely challenged (Ulenius et al., 2019).

#### Taurine-alcohol interactions

Taurine is a sulfur-containing amino acid and is the most abundant intracellular amino acid in humans (Gaull, 1989). It helps with skeletal muscle contractile function and attenuates exercise-induced DNA damage. Taurine also has other biological and physiologic functions; antiarrhythmic, inotropic, and chronotropic effects; central nervous system neuromodulation; endocrine or metabolic effects; and antioxidant and anti-inflammatory properties (Lourenço and Camilo, 2002; Juliano et al., 2014). Some studies have found that taurine is released from the nucleus accumbens following alcohol exposure and potentially plays a role in increasing extracellular dopamine levels in nucleus accumbens (Ericson et al., 2006, 2011). Elevated extracellular taurine levels may also be required for alcohol to induce dopamine release in the nucleus accumbens (Ericson et al., 2011). A study looking at the effect of taurine on alcohol-induced sleep time in mice found taurine to enhance the depressant effects of alcohol, suggesting an interaction between taurine and alcohol on the central nervous system (Ferko and Bobyock, 1988). Taurine may also influence some of the adverse effects of alcohol as shown by one controlled trial where pre-treatment with taurine reduced ethanol-induced increases of acetaldehyde in the blood and liver of rats (Watanabe et al., 1985). If this were the same in humans, it could be speculated that mixing EDs with alcohol means decreased hangovers with the same level of alcohol consumption. However, an opposing view is that the level of taurine and B vitamins (such as niacin) found in popular EDs are far below the amounts expected to deliver either therapeutic benefits or adverse effects (Clauson et al., 2008). A study exploring taurine and caffeine’s effect on ethanol-induced locomotion in mice failed to report any impact of acute administration of taurine on locomotion (Ulenius et al., 2019). The study does highlight an interesting finding where at particular dose combinations, co-administration of caffeine and taurine increased ethanol-induced locomotion to a greater extent than any drug administered alone or in combination (Ulenius et al., 2019). This contrasts with one previous study showing a dose-dependent relationship where taurine decreased ethanol-induced locomotion at low doses of ethanol but increased it at higher doses of ethanol (Aragon et al., 1992). This is still an area that is not sufficiently studied to make either claim and we hope future experimental research can try to build on this. However, despite the discrepancies, one can conclude that taurine may have subtle effects on ethanol-induced locomotion.

#### Glucuronolactone-alcohol interactions

Although glucuronolactone is one of the common ingredients in EDs, little to no research has been done on its health impacts in human or animal models. Additionally, there is no available literature on its interaction with alcohol, which is surely an area of interest. This was reiterated in studies published as early as 2010 (Higgins et al., 2010). Yet, over a decade later, no advancements have been made to try to understand this potentially harmful interaction. Despite many of these studies having been conducted on animal models and not replicated in humans, there is still concern over their effects on humans, even if not in identical mechanisms to that of the animal models.

### Potential adverse effects of alcohol mixed with energy drink and impact on safety and alcohol dependence

After briefly discussing the biochemistry that forms the basis of these interactions, we can begin to understand the effects they may have and their impact in humans, especially their most frequent users.

In addition to AMED being common practice in more than half of college students by some estimates (Malinauskas et al., 2007), it is also more common in binge drinkers compared to occasional consumers of alcohol. A survey on Michigan high school students in the US found binge drinkers; defined as drinkers who reported consuming five or more alcoholic drinks in a row during the 30 days before survey administration, to be more than twice as likely to mix alcohol with EDs when compared to non-binge drinkers (49.0 vs. 18.2%, *p* < 0.001) (Gonzales et al., 2015).

The reverse is also true, as frequent consumers of EDs are also more likely to consume more alcohol, and also at an earlier age. One study exploring the associations between caffeinated energy drink usage, alcohol-use patterns, and alcohol-related consequences in college students in the US, found that when compared to occasional ED consumers, weekly or daily ED consumers were more likely to have gotten drunk at an earlier age (Arria et al., 2011). High-frequency ED users were also found to consume alcohol more frequently and in higher quantities, and were twice as likely to be alcohol dependent than those who infrequently consume them (American Psychiatric Association, 2000). Another study also found that college students in the UK drank more alcohol on occasions when they also consumed EDs (8.6 drinks vs. 4.6 drinks; *p* = 0.016) (Price et al., 2010). This raises concerns over the lack of regulation over EDs which can potentiate dangerous interactions and propagate alcohol dependencies and binge-drinking given the existing moderate correlation. In addition to more frequent drinking, drinkers aged 15–23 who mix alcohol with EDs are also four times more likely to binge drink at high intensity when compared to drinkers who do not mix alcohol with EDs (Emond et al., 2014).

The effects of AMED can also have dangerous consequences wherein one health report studying the demographics of AMED in New South Wales, Australia found 39 poison center calls related to AMED recorded at NSWPIC (New South Wales State-Wide Poisons Information Centre). Over a 6 year period, there were 657 presentations related to AMED use across 59 emergency departments in NSW. The number of calls relating to AMED use were split evenly by gender and the majority involved adolescents and young adults. Over two thirds of poison center calls involved the co-ingestion of AMEDs with other substances as well (Lubman et al., 2013).

Literature has further highlighted the effects of AMED on binge-drinking where a measurement-burst design study looked at the short term consequences of AMED compared with consuming alcohol alone and found that AMED was associated with an increase in the number of alcoholic drinks consumed, more hours spent drinking, elevated blood alcohol content, and a higher probability of subjective intoxication (Patrick and Maggs, 2014).

Alcohol mixed with energy drinks is also associated with high risk sexual behavior and illicit substance use. A comparative study in the US looking at this association among young adults found AMED consumers to be significantly more likely to report marijuana, cocaine, and ecstasy use. They had higher odds of engaging in high-risk sexual behaviors such as unprotected sex, sex while under the influence of drugs, and sex after having too much to drink. This relationship remained significant after accounting for demographic factors and other substance use (Snipes and Benotsch, 2013).

A survey done in Canada that looked at whether youth who use AMED were more likely to engage in driving, or being a passenger of a driver under the influence of alcohol or cannabis compared to youth who use either alcohol or energy drinks alone, found youth who use AMED demonstrated a higher risk profile for driving under the influence of alcohol or cannabis than youth who use alcohol alone (Wilson et al., 2018). Additionally, alcohol and EDs both act as diuretics causing dehydration. Several deaths have been associated with ED consumption after sport as a consequence of dehydration (Finnegan, 2003). This poses serious concern, particularly in the younger population, where individuals are more physically active. Increased dehydration may also exacerbate the effects of a hangover, and lead to greater impairment the day after consumption (Finnegan, 2003). Isolated reports of death due to AMED have also been reported (Scimex, 2018).

The links, of varying strengths, between AMED and binge-drinking, high frequency and quantity drinking, earlier age of drinking and alcohol poisoning, and death, while may be of varying strength, are concerning enough to demand attention and stress the need to address and regulate this practice.

### Regulation of alcohol mixed with energy drinks around the world

We have explored the possible links, of varying strengths, between AMED and binge-drinking, high frequency and quantity drinking, earlier age of drinking and alcohol poisoning. While these may simply be correlations, they are concerning enough to demand attention.

In the early 2000s, beverages that combined alcohol, caffeine, and other stimulants, also known as caffeinated alcoholic beverages (CABs) were very popular (Federal Trade Commission, 2022; US Food and Drug Administration, 2022) but due to reported activities of alcohol poisoning, drunk driving, unprotected sexual intercourse and alcohol related injuries, the food and drug association (FDA) issued a notice requiring companies to remove CABs from the market in 2010. Standard alcohol and on-the-shelf caffeinated drinks do, however, remain available for individuals to mix, and regulations on EDs specifically are quite limited. The FDA lists caffeine, the primary ingredient in EDs, as “generally recognized as safe” when used in carbonated drinks at a certain level. Interestingly, this does not apply to EDs. The FDA has placed no restrictions on an upper caffeine limit in EDs whatsoever, meaning companies have no limitations over the caffeine content of their beverages and this is very concerning, given the potential for misuse (Heckman et al., 2010). Additionally, regulations in the US state that caffeine, along with any other ingredient, must be listed on the product label if added as an ingredient, however, the exact actual amount of caffeine, or any other ingredient, does not need to be listed on the label (Heckman et al., 2010).

Other regulatory bodies such as the Australia New Zealand Food Authority have a distinct category of beverages called “formulated caffeinated beverages” which must contain no less than 145 mg/L and no more than 320 mg/L of caffeine, which includes all caffeine present, regardless of the source (Heckman et al., 2010). Restrictions also exist for the amount of taurine and glucuronolactone permitted in EDs. The only regulation on the supply of AMEDs in Australia, is in Perth, Western Australia, where venues are prohibited from selling AMEDs after midnight, but this policy is undermined by the continued sale of EDs in the same venues after midnight (UK Wired, 2010). The European Union on the other hand, has not set an upper limit for caffeine content; however, if the beverage contains more than 150 mg/L, the product label must read “high caffeine content” followed by the amount of caffeine (Heckman et al., 2010; Lubman et al., 2013). In the UK, regulations are yet to be implemented. A proposed ban was resisted by Scotland in 2005 (UK Wired, 2010).

There is some progression in the regulation of this practice over the last few years. Regulatory authorities in Canada restrict the manufacture and sale of CAB unless the caffeine is derived from a natural source such as guarana, however, caffeine as an ingredient cannot be directly added to an alcoholic drink (UK Wired, 2010). The authorities have also recently reduced the amount of caffeine in EDs (no more than 400 mg of caffeine per liter or 180 mg per single serve) and EDs are now required to display the following warning labels: “do not mix with alcohol,” “high source of caffeine,” and “not recommended for children, pregnant/breastfeeding women, individuals sensitive to caffeine,” In Mexico, regulatory bodies have prohibited the sale of AMEDs in licensed venues (Lubman et al., 2013).

## Conclusion

This review sheds light on the effects AMED can have from various perspectives. These include, but are not limited to, binge-drinking, more frequent and higher quantity consumption, younger age at drinking, alcohol poisoning, more negative after-effects, as well as engaging in risky sexual behavior and illicit substance use. The active ingredients in EDs contribute significantly and are part of the mechanism by which these effects are significantly increased in those mixing EDs with alcohol compared to those consuming alcohol alone, although there remain uncertainties on the evidence surrounding the exact mechanisms underlying interactions between alcohol and energy drink constituents. Further human studies of the interaction between alcohol and caffeine are needed to confirm the findings of animal models, while both animal and human studies are lacking for other ED ingredients.

However, in spite of these uncertainties on interactions, there is still a body of evidence suggestive of risky behaviors and harmful effects in people who consume AMED, underlining the public health need for regulation of the widespread global consumption of these. Further research in this area would be beneficial and aid regulatory bodies in the making of guidelines regarding the use and effects of AMED in years to come.

## Author contributions

JS, JP, and HC: literature review, writing, editing, and revision of the manuscript. All authors contributed to the article and approved the submitted version.
